# Occipital Nerve Blocks in the Emergency Department for Initial Medication-Refractory Acute Occipital Migraines

**DOI:** 10.5811/cpcem.2019.1.39910

**Published:** 2019-01-22

**Authors:** Justin Yanuck, Sagar Shah, Maxwell Jen, Rakhi Dayal

**Affiliations:** * University of California, Irvine, Department of Emergency Medicine, Orange, California; † University of California, Irvine, Department of Anesthesiology, Orange, California

## Abstract

Migraines are consistently among the top 20 primary coded diagnoses in emergency departments, constituting 4.5% of all chief complaints. In a significant subset of these, pain arises from the occipital region innervated by the greater (GON) and lesser occipital nerve. In this case series, we present three patients with occipital migraines who received GON blockade with 1% lidocaine. The blockade was performed only after first-line treatment with metoclopramide and possibly additional medications as ordered by triage physician, failed to adequately alleviate pain by 40 minutes after medication administration. Patients were contacted a minimum of seven days following treatment. All three patients experienced significant analgesia and relief of symptoms within 15 minutes of blockade and sustained relief through a seven-day follow-up period.

## INTRODUCTION

Up to 4.5% of emergency department (ED) visits carry a chief complaint of non-traumatic headache.^1^ A subset of these headaches arise from the occipital region and are diagnosed as occipital migraines. The occipital nerve (of which the greater and lesser occipital nerves make up the primary sensory fibers) innervates the posterior aspect of the head to the vertex.^2^–^4^ While the etiology of an occipital migraine is unclear, there is evidence that local nerve blocks of the greater occipital nerve (GON) and the lesser occipital nerve (LON) contributes to analgesia by interrupting the facilitatory effect of GON stimulation on central pain sensitization of second-order neurons.^5^,^6^ Although there are currently multiple treatment options for occipital migraines, there is vast heterogeneity in treatment patterns among emergency physicians.^7^ While a majority of patients still receive opioids and non-steroidal anti-inflammatory medications for acute migraines, there is data to support that anti-dopaminergics (such as metoclopramide) offer superior therapeutic benefit with the most benign side-effect profile.^8^,^9^ However, further treatment modalities remain desirable given that even with metoclopramide, there is incomplete pain relief and high recurrence of migraines within one month.^10^ In this case series, we describe three distinct patient cases who presented to the ED with occipital migraines that had incomplete resolution of pain following initial home abortive medications and then a 10 mg dose of metoclopramide, who subsequently underwent bedside occipital nerve block which achieved significant improvement in pain. We subsequently followed-up with these patients one week after the ED visit to evaluate their post-treatment symptom course.

## CASE SERIES

### Case 1

A 17-year-old female with history significant for twice-weekly migraines presented to the ED with 12 hours of a persistent right-sided occipital migraine described as constant, sharp, and 10/10 in severity. The patient had associated nausea, with no other neurologic symptoms, and no recent fever or head trauma. The patient took acetaminophen and sumatriptan at home, which helped for two hours, before subsequent recurrence. On exam, the patient had mild right occipital tenderness to palpation, with no midline spinal tenderness and no neurologic deficits. The patient received metoclopramide 10 mg in triage and one liter of normal saline. Approximately 40 minutes after initial treatment, the patient noted her pain had improved from 10/10 to 8/10 severity. The patient then received one milliliter (mL) injection of 1% lidocaine 1cm to the right GON. Approximately 60 minutes after medications had been given, and ten minutes after occipital nerve block, the patient noted her pain improved to 2/10. During follow-up phone interview at seven days, the patient noted her symptoms completely resolved one hour after discharge, and that over last seven days she had not had any further migraines.

### Case 2

A 48-year-old female presented to the ED with three days of persistent bilateral occipital pain that was constant, sharp in quality, and was 8/10 in severity. The patient had past medical history only significant for hyperlipidemia and migraines. The patient usually suffered one to two migraines per month. In addition to her headache, the patient also endorsed nausea and three episodes of emesis. She took sumatriptan, acetaminophen, and ibuprofen in the 48 hours prior to arrival with minimal relief. The patient denied any other symptoms. On exam, the patient was noted to have mild bilateral occipital tenderness to palpation and no neurological deficits or midline tenderness. In ED triage, the patient received metoclopramide 10 mg and ketoralac 15 mg intravenously. Approximately 60 minutes after the patient received these medications she was re-assessed and found to have persistent head pain rated at a 7/10 in severity. Bilateral GON blocks were administered with a total of one mL of 1% lidocaine to each site. At 15 minutes and 1.5 hours post-procedure, the patient reported pain improvement to 3/10. During follow-up phone interview at nine days post-emergency department visit, patient noted her pain had resolved over the course of 24 hours, with no recurrence of a migraine.

### Case 3

A 37-year-old male presented to the ED with past medical history significant for anxiety and once monthly migraines. The patient described the pain as originating from the back of his head and radiating forward. The pain was located only to the right side, was constant and sharp in nature, and rated at a 10/10 in severity. The patient had the pain for 12 hours. The patient noted that the pain was typical for his migraine; however, his typical home abortive medication, ibuprofen, did not work for him on this occasion. The patient also tried one hydrocodone/acetaminophen 5/325 three hours prior to arrival (which he had obtained during previous emergency department visits for the same head pain) but without improvement. The patient denied any recent head trauma, fevers, or neurological deficits. On exam, the patient had no midline spinal tenderness, no motor/sensory deficits, or cranial nerve abnormalities. The patient was noted to have right occipital tenderness to palpation. The patient was given metoclopramide 10 mg, one liter of normal saline, and diphenhydramine 25 mg by the ED triage physician. Approximately 45 minutes after the medications were given, the patient was re-assessed and stated his pain had improved from a 10/10 to an 8/10. The patient then received one mL of 1% lidocaine to the right GON. Approximately 60 minutes after the patient received the initial medications, and three minutes after the patient received the occipital nerve block, the patient reported the pain had improved to 2/10. Follow-up phone call interview conducted at day eight revealed that the patient’s migraine never recurred. The patient noted that his symptoms had completely resolved following the injection and that if he had a migraine again, he would preferentially seek out an occipital nerve block.

**CPC-EM Capsule**

What do we already know about this clinical entity?

*Migraines are among the top 20 coded diagnoses in emergency departments, constituting 4.5% of all chief complaints*.

What makes this presentation of disease reportable?

*These cases present a simple bedside procedure that is not well known or practiced in the emergency department*.

What is the major learning point?

*Occipital nerve blocks are a possible treatment modality for occipital migraines*.

How might this improve emergency medicine practice?

*This report provides an additional tool to treat refractory migraines which is critical to improving outcomes, reducing costs, and providing quality care for patients*.

## DISCUSSION

In this case series, we highlight three patients who presented to the emergency department with occipital migraines who failed to receive adequate relief of symptoms from initial conventional therapy. We demonstrate that a simple bedside procedure that can be performed by all emergency physicians can easily and safely provide patients with significant relief from treatment-refractory occipital migraines and with sustained relief during a seven-day follow-up period. The above three patients all had migraines or probable migraines without aura as defined by the International Headache Society’s International Classification of Headache Disorders (ICHD) with a self-reported majority of pain in the occipital region.^11^ It is this subset of occipital migraine patients for whom we sought to elucidate whether occipital nerve blocks could provide relief of symptoms to initial treatment refractory pain.

Anesthetic nerve blockade for an occipital migraine is thought to exert its effects via modulation of cervical nociceptive signals that converge on the spinal trigeminal nucleus caudalis and subsequently travel to higher cortical structures. Both the GON and LON travel back through the second cervical vertebrae (C2) spinal nerve through the dorsal ramus (greater) and ventral ramus (lesser.) Nerve block studies for cervicogenic headaches found GON blockade to be as effective as complete C2 spinal nerve blockade^12^, suggesting it makes little difference as to whether the pain is mediated by the GON or the LON. Given the relative ease of a single GON injection, we suggest GON blockade alone is sufficient for initial treatment of an occipital migraine in the ED.

While multiple techniques exist for the performance of the occipital nerve block, many involve a fanning technique and addition of some form of a steroid; there is no consensus as to what provides the most effective immediate and long-term relief of symptoms.^13^–^16^ For this case series, we chose to use a simple technique that requires only one needle insertion and does not include steroid. Either the left, right, or bilateral sides were prepped with a sterile alcohol pad. Utilizing a 27 gauge 1–3/8 inch needle, 1 milliliter of 1% lidocaine was injected at 90-degree angle to skin immediately medial to the occipital artery with care taken to target the occiput above the intermastoid line (the imaginary line between the external occipital protuberance and the mastoid process). The needle is inserted until it hits bone, which is usually about 1 centimeter and then withdrawn slightly off of the bone before infiltration with the lidocaine. This anesthetizes the GON.^17^ If the occipital artery could not be detected by palpation, the injection was made 1–2 cm lateral to external occipital protuberance given that the GON typically lies 1–2 cm lateral to the external occipital protuberance (Figure, Video).

Migraines are consistently among the top 20 primary coded diagnoses in emergency departments.^18^ Furthermore, the 2007 Centers for Disease Control and Prevention ambulatory medical utilization estimates found that 18% of all migraine care occurs in an emergency department setting.^18^ Given migraines are just one of many clinical diagnoses for which opioids might be indicated, ED physicians are the most frequent “first-prescribers” of opioids.^19^ While in this case series no ED provider prescribed opioids as initial therapy for these patients’ occipital migraines, it is our experience that for many patients who experience initial treatment-refractory migraines, many ED providers will utilize opioids as the next treatment modality. In fact, a 2017 study found that opioids were prescribed in 36% of migraine diagnoses in three diverse emergency departments. In 30% of those cases, those opioids were given as first-line treatment. Alternatively, in 49% of those cases, opioids were given as rescue therapy (within 60 minutes of initial diagnoses) when initial treatment was ineffective.^20^ Although there is variation in opioid prescription rates by practice setting, these findings are not reflective of the guidelines recommending against opioids for migraines. Providing emergency physicians with an additional tool to effectively treat refractory migraines without opioid therapy is critical to improving outcomes, reducing costs, and providing quality care for patients.

The use of triptans, beta-blockers, anticonvulsants, antidepressants, and opioids for the treatment of migraines may have unsatisfactory efficacy or undesirable systemic effects.^10^,^21^–^22^ In the three cases presented here, the patients came to the ED because abortive therapy was ineffective. Beyond failing to reduce the pain during initial presentation, these medications may be poorly tolerated long-term; furthermore, noncompliance or misuse may lead to the chronification of migraines. Prior data indicates that one in five patients will discontinue preventative medications for tolerability or safety concerns.^21^ Less than a quarter of patients prescribed oral preventative medications will remain compliant for more than 12 months following initial treatment.^22^ The management of treatment-refractory migraine challenges emergency physicians to provide adequate pain relief, minimize time spent in the ED, prevent repeat ED visits, and minimize the risk of substance abuse. As shown here, occipital nerve blocks possibly provide a tool that could address some of these concerns.

## CONCLUSION

The three cases presented here suggest occipital nerve blocks could potentially be used to alleviate treatment-refractory migraines in the ED. Often times, when a first-line therapy, such metoclopramide which was given to these patients, fails to alleviate migraine pain, many emergency departments use opioids as rescue therapy.^18^ Although this small case series does nothing to formally establish the efficacy of occipital nerve blocks for initial treatment refractory occipital migraines, it does highlight a useful tool that could be tried by an emergency physician. Current evidence for the effectiveness of occipital nerve blocks in the management of chronic migraines justifies the additional study of occipital nerve block use for acute occipital migraines that present to the ED. Future direction includes a prospective randomized controlled study to assess the role of this technique in patients presenting to the ED with a refractory occipital migraine.

## Supplementary Information

Video Demonstration of occipital nerve block for the treatment of refractory occipital migraine.

## Figures and Tables

**Figure:**
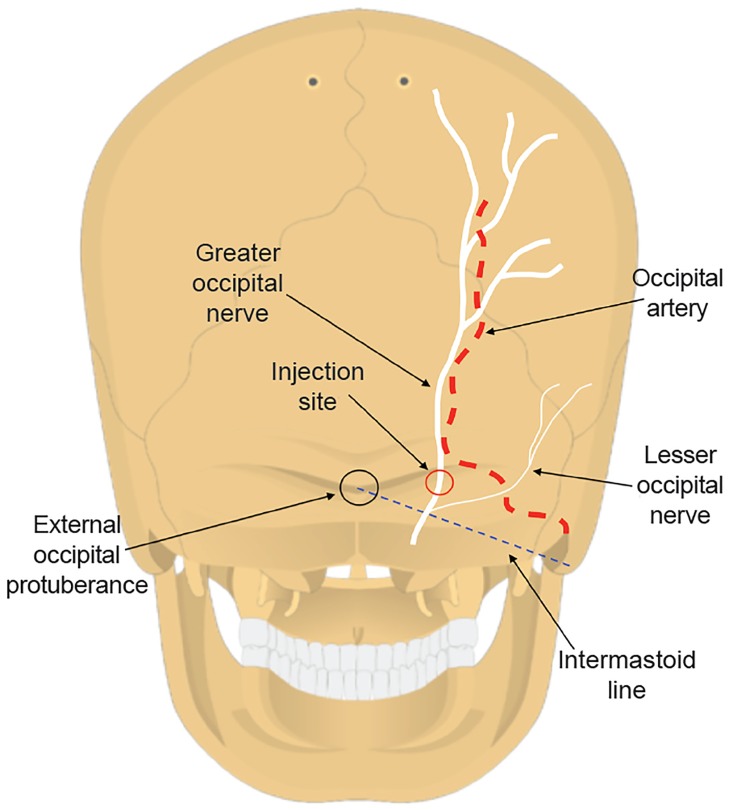
The diagram demonstrates the location of the intermastoid line, external occipital protuberance, greater occipital nerve, and lesser occipital nerve. The injection site is located just medial to the occipital artery and should be targeted above the intermastoid line. The needle is inserted until it hits bone and retracted slightly off of the surface.
